# Exercising alcohol patients don’t lack motivation but struggle with structures, emotions and social context - a qualitative dropout study

**DOI:** 10.1186/s12875-017-0606-4

**Published:** 2017-03-23

**Authors:** Sengül Sari, Ashley Elizabeth Muller, Kirsten K. Roessler

**Affiliations:** 1 Department of Psychology, University of Southern Denmark, Campusvej 55, 5230 Odense, Denmark; 2 Unit of Clinical Alcohol Research (UCAR), Institute of Clinical Research, University of Southern Denmark, Odense, Denmark; 3 Norwegian Centre for Addiction Research (SERAF), Institute of Clinical Medicine, University of Oslo, Oslo, Norway

**Keywords:** Alcohol use disorder, Participation, Best practice, Dropout, Outpatient treatment, Physical exercise, Relapse prevention

## Abstract

**Background:**

Exercise is an important component of a healthy lifestyle, the development of which is a relapse prevention strategy for those with alcohol use disorder. However, it is a challenge to create exercise interventions with a persistent behavioural change. The aim of this qualitative study was to investigate perceived barriers to participation in an exercise intervention among alcohol use disorder patients, who dropped out of the intervention program. Furthermore, this study aims to propose possibilities for a better practice of future intervention studies based on the participants’ experiences and suggestions.

**Methods:**

Qualitative interviews with 17 patients who dropped out from an exercise intervention in an outpatient treatment centre about their experiences and reasons for dropping out. Social cognitive theory informed the development of the interview guides and systematic text condensation was used for analysis.

**Results:**

Analysis revealed three central themes: 1) Structural barriers described as the type of exercise and the timing of the intervention, 2) Social barriers described as need for accountability and unsupportive relations, and 3) Emotional barriers described as fear, guilt and shame, and negative affect of the intervention on long term.

**Conclusions:**

Future exercise interventions should include socio-psychological support during the first weeks, begin shortly after treatment initiation instead of concurrently, and focus on garnering social support for participants in both the intervention context and among their existing network in order to best reduce barriers to participation.

**Trial registration:**

This study was retrospectively registered at Current Controlled Trials ISRCTN74889852 on 11 July 2013.

**Electronic supplementary material:**

The online version of this article (doi:10.1186/s12875-017-0606-4) contains supplementary material, which is available to authorized users.

## Background

Exercise is an important public health recommendation to the general public, clinical groups, and across the lifespan, as physical inactivity is the fourth leading risk factor for global mortality [1]. General practitioners often have first access to persons who are physically inactive and at risk for chronic diseases, and GP referral to exercise programs and specialists is a relatively recent strategy [1–3]. In addition to reducing the risk of conditions such as hypertension, diabetes, depression, and cardiovascular disease, exercise has also been implemented as an adjunct treatment itself for many chronic diseases, including substance use disorders such as Alcohol Use Disorder (AUD). Exercise is a promising strategy in alcohol relapse prevention through psychological mechanisms, such as reducing negative affect and depression and creating pleasurable states, through reducing stress reactivity, and by reducing cravings [4]. Additionally, exercise is one of several lifestyle modifications, suggested as a relapse prevention strategy in alcohol recovery and coping strategy in high-risk situations [5].

As health care providers are well aware, maintaining an exercise program is the next hurdle after beginning, because persistent behavioral change - i.e., continued activity after the course of the program - cannot be incited if participation is not maintained. High drop-out rates are therefore of concern, and barriers to participation have been investigated in numerous qualitative and quantitative studies, however only few recent studies reported suggestions to better physical exercise interventions for AUD and other substance use disorder patients [6–10]. Especially Kendzor et al. [6] and Muller and Clausen [7] suggest involving participants in the design phase to create a feasible structure of the interventions. For exercise interventions in general, most common barriers to physical activity are psychological factors such as lack of motivation, low self-efficacy and low fitness expectations, while environmental barriers to physical activity include lack of time, transport and financial costs [11]. Hence, Read et al. [8], Stoutenberg et al. [9] and Abrantes et al. [10] suggest that the alcohol treatment field could benefit from existing knowledge of intervention designs and motivational techniques from the larger exercise science field to improve adherence to interventions specifically targeted individuals with AUD.

Social factors deserve further attention when designing exercise interventions, as social support forms what Ntoumanis and Biddle call the “motivational climate” [12]. Social integration with friends and family predicts higher probability of meeting physical activity guidelines and lower probability of inactivity [13], which could partially explain the association between social isolation and chronic disease. Persons with AUD are both less physically active [10] and more socially isolated [14] than those without AUD, and more severe diagnoses are associated with poorer social networks [15]. It is therefore likely that individuals with AUD are even more challenged in finding and maintaining the motivation to exercise. An answer to how these individuals can best be supported was sought among dropouts from the Healthy Lifestyle Study, a randomized controlled trial of exercise for persons receiving outpatient AUD treatment in Denmark [16], and informed by social cognitive theory [17].

This paper focused on the patients who dropped out of the Healthy Lifestyle Study. The aim of this qualitative study was to investigate perceived barriers to participate in an exercise intervention among alcohol use disorder patients, who dropped out from the Healthy Lifestyle Study. Furthermore, this study aimed to propose possibilities for a better practice of future intervention studies based on the participants’ experience with the Healthy Lifestyle Study and their suggestions for alternatives to the intervention. The advantages of using a qualitative approach is to evaluate dropout reasons and intervention designs in depth and in detail where data depends on human experience and this is more compelling and powerful than data gathered through quantitative research [18].

## Methods

### Theoretical framework

To understand and describe the theoretical aspects of exercise as a health behaviour that is affected by several mechanisms surrounding the individual, we used Bandura’s Social Cognitive Theory [17] as a framework, which has successfully been applied in studies exploring exercise behavior in other chronic disease and rehabilitation groups [19, 20]. Social cognitive theory directed the design of the semi-structured interview guide used in this study which aimed at answering our research question: What were the reasons for dropping out of the exercise intervention, and how should an exercise intervention be like for AUD-patients to participate in a persistent way?

The understanding of the capacity to anticipate and place value on the outcome of different behaviour patterns emphasizes the importance of understanding personal beliefs and motivations underlying different behaviour. Participants in this interview study were therefore asked about their experiences with the intervention and with exercise in general.

We hypothesized that participants in an exercise intervention would be more likely to uptake and maintain a healthier lifestyle if they had relations who were regular exercisers and appreciated a healthy lifestyle, whom they regarded as sophisticated and attractive. If they observed and valued the rewards that they associated with exercising, such as a desirable self-image or wellness, then they would be more likely to exercise themselves. Such an understanding further reinforces the importance of taking account of peer influences and social norms on health behaviour [21], and of the potential use of role models in influencing social norms [22]. Furthermore, development of social support through participation in especially group-based exercise interventions which have the potential to foster interpersonal relationships may also contribute to recovery [23]. Social support to exercise and social support to participation in an exercise intervention were therefore essential elements which we wished to explore in this study.

Social cognitive theory emphasizes the interactions between an individual and their environment, and suggests an environment can shape behaviour by making it more or less rewarding to behave in particular ways [17]. In the setting of an exercise program, an environment in which others co-exercise and are positive towards facilitating the individual’s exercise could be less rewarding for the individual who does not exercise. The Healthy Lifestyle Study therefore randomized participants into an individual exercise program or a group exercise program, in order to understand if participants were influenced by the social environment created by peers and supervisors in the group sessions. Social cognitive theory operates with observational learning which describes the capacity to learn by observing both the behaviour of others and the rewards received for different patterns of behaviour. The significance of role models is essential here.

An understanding of this interaction and the way in which modification of social norms can impact behaviour offers an important insight into how behaviour can be modified through health promotion interventions. This interaction is further supported by the findings of a study of mediators of physical activity behaviour change, where changes in behavioural processes and exercise-induced feelings were found to satisfy the theory of treatment effects on mediators and the theory of mediator effects on physical activity [24].

### Participants and setting

Participants in this paper were drawn from the Healthy Lifestyle Study was implemented in an outpatient treatment centre in Denmark to evaluate the effect of exercise as adjunct to treatment-as-usual of AUD. The study population consisted of 175 consecutively admitted patients who were randomized to either treatment-as-usual or one of two 6-month interventions that were selected on the basis of existing evidence-based studies [25, 26]. In the first intervention group, participants exercised individually after receiving basic instructions and a training program for home use. In the second intervention group, several patients exercised together with two instructors in 60-min training sessions twice a week. Running was the specific exercise form for both groups in the study. The main study outcomes were fitness, mood and drinking behaviour. The inclusion criteria for participating in the Healthy Lifestyle Study were: diagnosis of AUD, abuse or dependence according to ICD-10, age over 18 years, Danish speaking, without severe psychosis or cognitive impairment, and without severe physical disabilities or medical problems which would inhibit exercise.

A total of 65 of the 175 included patients in the Healthy Lifestyle Study dropped out during the 6-month intervention period, either immediately after the randomization or over time. Drop out was indicated when participants directly reported to project personnel or when they ceded participation without contact. There were no differences in age, gender, education, or other demographics between those who dropped out and those who completed. Participants gave their acceptance to be contacted for a “dropout interview” at inclusion. Of the 65 who dropped out, 17 agreed to participate, of which four were women and 13 were men, nine were allocated to the group intervention, seven were allocated to the individual intervention and one were allocated to the control group. Participants were aged between 30 and 68 years. Their length of participation before dropout varied from 2 days to 12 weeks. Names mentioned in quotes are fictive due to anonymization.

### Data collection

We pilot-tested an interview guide (see also Additional file 1: Semistructured interviewguide English.docx) for use in semi-structured interviews by interviewing one of the participants who dropped out after 1 week. After the pilot interview we adjusted the interview guide in order to ask questions in a more natural and meaningful way, and at the same time allowing the respondent to lead and form the conversation. The interview guide was developed on the basis of the research questions [27].

All interviews were conducted face-to-face in an undisturbed setting at the outpatient clinic. Participants were asked open-ended questions in a way that allowed them to answer reflectively, in turn giving the interviewer the possibility to ask follow-up questions in more depth. Interviews were conducted by the project leader or the instructors of the exercise intervention, whom participants had previously met. The duration of the interviews was 14–30 min, and all were audio-recorded and transcribed verbatim.

### Analysis

The strategy for qualitative analysis was systematic text condensation [28], similar to qualitative content analysis [29], a technique in which the expressed meanings of the respondents are given shorter formulations but understood as presented by respondents, rather than the researcher attempting to search for unexpressed, underlying meanings. It involves four steps, all of which were conducted by the authors SS and KR: transcripts were read to obtain a general sense of the data; natural meaning units as expressed by participants and as pertinent to our themes of exercise, participation in the exercise intervention, and social support, were identified; the dominant themes of natural meaning units were reformulated to be more direct; and these themes were linked in descriptive statements [28]. Systematic tense condensation is developed on the basis of phenomenological philosophy and is used in a range of qualitative research [30, 31]. This methodology allows the researcher to work systematically with data expressed in ordinary language and to show stringency and discipline in data analysis without converting data to quantitative expressions.

## Results

Analysis of the interviews moved from natural meaning units to three central themes as reasons for dropout or barriers to participating in the intervention, as illustrated in Table 1: 1) *Structural barriers,* described as the type of exercise and the timing of the intervention, 2) *Social barriers,* described as need for accountability and unsupportive relations, and 3) *Emotional barriers,* described as fear, guilt and shame, and negative affect of the intervention after some time. A more detailed description of the central themes is presented in the following with quotes from the interviews. Participants’ suggestions for future interventions and participants’ alternatives to the exercise intervention which were revealed during the interviews are also presented.

**Table 1:** Process from natural meaning units to development of the central themes by using systematic text condensation

Natural meaning units	Themes	Central themes
Running is boring	Type of exercise	Structural barriers
Running caused pain in my knee and back		
I prioritized my family and job	Timing	
The treatment itself was enough time and energy consuming		
I needed somebody to encourage me	Need for commitment	Social barriers
Running with others would motivate me		
There was no feeling of communion in the group		
I needed support from the group members	Unsupportive relations	
My family did not support me		
I have no family		
I feared that I could not perform as well as the others in the group	Fear, guilt and shame	Emotional barriers
I used to look good		
I was used to exercising a lot in the past, but not anymore		
The intervention reminded me of my alcohol problem	Negative affect	
I don’t need help anymore		

### Structural barriers and replacements

Running or jogging was neither the most preferred nor most enjoyed type of exercise for many of the participants. Often due to past injuries, it was painful or simply boring, and they therefore stopped.

Some participants mentioned that the timing of the project was not appropriate. Although the Healthy Lifestyle Study was designed as a supplement to treatment-as-usual, some of the participants were not ready to do anything else than the treatment itself at that time. They had conflicting feelings and sometimes depressive symptoms to such a degree that they could not find any mental resources to start up anything else than the traditional treatment. Once patients initiated treatment they discovered issues that they had long neglected, such as within their families and jobs, and they were now ready to confront those issues, but not in tandem with the intervention.

*“I had so many other things to think about […]. They occupied my mind very much. I didn’t feel that I had the mental resources or power to be a part of this also. I couldn’t manage it”. (Bo)*

Instead, they suggested offering exercise interventions 1 month after treatment initiation or as an after-treatment.

### Social barriers

Different barriers were expressed by participants with little or no former exercise experience compared to those who were comfor exercising based on previous experience. Inexperienced participants expressed a substantial need for social support to uptake and maintain a participation. They preferred to exercise in groups, or at least with one “contact person” to encourage them before exercising and accompany them during exercising. As described, the contact person could also act as a source of accountability. Feelings of reliability were often raised by participants who preferred to exercise with others, and it was a lack of this accountability that led many inexperienced exercisers who were randomized to individual training to drop out from the study. In contrast to this, peer support was enjoyable for experienced exercisers, but not necessary, which is why they often maintained exercise independently after dropping out.

The importance of supportive social relations was pointed out by participants who had unsupportive relations or no relations at all. They were all physically inactive and dropped out due to lack of family support. That their motivations toward the exercise intervention were not supported by their social relations became demotivating, and eventually they dropped out.

The participants had different needs of the social environment of the intervention. Some of them wanted to share their treatment experiences with peers. This need was not fulfilled in the intervention either because of being randomized to the individual arm, or because a feeling of communion was not established in the group. Some group participants reportedly attended only for the exercise, and their peers who wanted to discuss alcohol issues in an informal setting experienced this as a disappointment and eventually dropped out, fulfilling this need at other places such as Alcoholics Anonymous.

### Emotional barriers

Another reason to drop out from the group intervention was fearing that others would be fitter than oneself. There was an underlying feeling of guilt and shame over their fitness levels which were hard to overcome, especially if the participant was used to exercise at high levels earlier in life. Feeling overweight and unattractive was also a barrier towards exercising in groups because of a fear of embarrassing oneself in front of others. Conversely, mirroring functioned positively for inexperienced exercisers who perceived other participants in the group as similar to themselves regarding performance level and physical appearance. They were less likely to cite emotional barriers, and these participants had more positive expectations of the outcome of participating in the group intervention.

For others, participation itself labelled them as “alcoholic”. They dropped out after ending treatment because they no longer needed help to recover from AUD, and the intervention affected them negatively by reminding them of their former alcohol problem. Everything related to AUD treatment, inclusive of the exercise intervention, belonged to the past and at a certain point in time there developed a desire to move forward.

*“One has to be careful with not getting stuck, and that it does not become too much of one’s identity. That was how I felt after Christmas so I stopped coming […] You also have to move on a little in life, right. There has to be a balance. You shouldn’t keep hanging on your own misery, and on the other side, you shouldn’t neither keep thinking new possibilities”. (Chris)*

*“I just don’t want to be reminded of the alcohol thing, because I actually think it’s over”. (Dorothy)*

## Discussion and recommendations

In this qualitative study, we interviewed 17 patients who dropped out of an exercise intervention adjunct to outpatient treatment for an alcohol use disorder (AUD). However, participation, even when brief, conferred two benefits: it gave them an example of how exercising could function as a replacement of alcohol in their lives, and they found their own alternatives after dropping out, such as cycling, swimming, and going to a fitness centre. Second, it taught and reminded them of the positive mental benefits of exercise, expressed as happiness, renewed energy and concentration, less stress, wellness and relaxation. These anticipated effects provided inspiration to adopt a more physically active lifestyle; for example, beginning to cycle for transportation or finding a training partner in their social networks.

Dropping out of the exercise intervention was mostly not connected to lack of interest or motivation to exercise. Rather, reasons expressed by participants were divided into *structural, social,* and *emotional barriers*, many of the same pointed out in the literature among healthy and other clinical populations [32–34]. Our findings are also similar to those reported by physically inactive AUD patients [8, 9], and by a more heterogeneous substance use disorder patient group [10], suggesting that the alcohol treatment field could utilize existing knowledge of intervention designs and motivational techniques from the larger exercise science field to improve participant retention. The recommendations made here are based on our participants’ suggestions to ideal exercise interventions which they expressed as they would be adherent to.

The intervention structure did not match participants’ schedules or exercise preferences, and it was not possible for the intervention be flexible. An important improvement to future interventions would be involving potential participants in the design phase in order to create a more *feasible structure*, which, along with flexibility, was also a best practice identified by a pilot exercise program for participants with heterogeneous substance use disorders [7]. However, it is positive that participants were able to overcome these structural barriers not to participate in the intervention, but to exercise in other ways: many participants were inspired to a healthy lifestyle via the intervention and implemented alternative behaviors in their everyday life. Our findings agree with Kendzor et al’s [6] recommendation that physically activities already endorsed by persons with AUD should be the activities incorporated into interventions.

Additionally, the importance of *social support and social influence* in behaviour change, as described in the social cognitive theory, are also some of the essential themes which we recognize in our findings. Previous studies suggest that individuals who are less personally attracted to the group’s task and to the group as a social unit are more likely to drop out from an exercise intervention than individuals who are more attracted to the group [11]. Experienced exercisers in this study, therefore, would have needed a higher attachment to the group’s social unit to compensate for their attachment to the group’s task, which was low because the level of exercise was suboptimal for them. Conversely, inexperienced individuals needed others to get motivated to exercise, to function as role models to support their observational learning, and to provide accountability. Inexperienced participants were more attracted to both the group’s task and to the group as a social unit. It is unsurprising that inexperienced participants who were randomized to individual exercise did not find motivation to proceed, as they lacked social support. Roessler et al. [35] argue that supportive relationships within intervention groups may be beneficial for establishing a healthy lifestyle. This particular argument is also significant in our study where a lack of a feeling of communion with peers played an important role for the participants’ lack of cohesion to the group. Future exercise interventions for AUD-patients may therefore benefit from focusing on creating group cohesion and opening up for sharing alcohol-related experiences. In this way it can be possible to promote both observational learning and participatory learning for behaviour change.

Another social phenomenon of the group intervention was participants using each other as mirrors. Experienced exercisers who were dissatisfied with their current condition and appearance dealt with feelings such as guilt and shame, because they expected these aspects to be inferior to their group members. Dropping out was a way to avoid guilt and shame, and was also a result of having low expectations of the outcome, such as the intervention not being able to benefit their fitness levels. Body image concerns are not limited to participants of this intervention: Brudzynski and Ebben [36] reported the same avoidance of group settings among college students with body image concerns.

In general, a group structure is more recommended than an individual program, but we recommend that homogenous groups may be the most effective. This indicates that homogenous groups may be effective in future interventions.

Having supportive relations outside of the intervention was a specific motivator to be physically active, and participants with unsupportive and/ or physically inactive relations struggled more, similar to Cole et al.’s [37] qualitative findings among patients with coronary heart disease. Particular attention in future interventions should be paid to participants’ existing social networks and those networks’ levels of exercise. As Anderson et al. [38] suggested, increasing social support to exercise may be key to increasing the individual’s self-efficacy and self-regulation skills necessary to exercise. Extra support and encouragement to attend sessions could be given to those who have little experience with exercise and who lack examples of exercise. For example, extra SMS reminders to attend exercise sessions or setting short-term, achievable goals could be useful.

### Intervention as a label of AUD

The intervention was identified as a reminder of having an AUD, and for those who no longer identified as having a problem, participation was not worth keeping this label. The duration and setting of exercise interventions is therefore worth considering in future studies. This begs the question of whether brief exercise interventions could be more useful in preventing dropout caused by negative affect. Another solution could be selecting a neutral setting for the interventions which does not remind participants of present or past AUD treatment. Exercise sessions in this study were located at the AUD treatment centre and this may have led to negative affect on long term.

## Conclusion

Participants in AUD treatment who dropped out from an adjunct exercise program did not so primarily because of lack of motivation per se, but because they struggled with many of the same *structural*, *social*, and *emotional* barriers reported by other groups. One important recommendation they made is for future programs to utilize a group structure where members have similar performance levels and where peer-support enhances group cohesion, thus increasing adherence. It is also important for intervention designs to be more easily integrated into participants’ lives and contain more varied and interesting activities.

## Additional file

Additional file 1: Semistructured interviewguide English. Interview guide for the dropout study. Description: Research questions and interview questions used for the study of dropouts from an exercise intervention for patients in treatment for alcohol use disorder. (DOCX 35 kb)

## Abbreviation

AUD: Alcohol use disorder
