# Supplementary material for: Exercising alcohol patients don’t lack motivation but struggle with structures, emotions and social context - a qualitative dropout study
Source: BMC Fam Pract. 2017 Mar 23;18:45. doi: 10.1186/s12875-017-0606-4 (PMC5363022; doi:10.1186/s12875-017-0606-4)
Supplement: Additional file 1: — Semistructured interviewguide English. Interview guide for the dropout study. Description: Research questions and interview questions used for the study of dropouts from an exercise intervention for patients in treatment for alcohol use disorder. (DOCX 35 kb) [file 12875_2017_606_MOESM1_ESM.docx]

**Interview guide for the dropout study**

| Research questions | Interview questions |
| --- | --- |
| Former experience with exercise | - What are your former experiences with physical activity? - What does exercise mean to you in your daily life? |
| The importance of social relations regarding physical activity for each individual | - How do you experience support from your family and other relations regarding doing exercise? - If job: How does your workplace make it possible for you to participate in the study and to exercise in general? |
| Expectations to the study | - What were your expectations to the study at the time when you were introduced to it? - What are the reasons for you to no longer participate in the study? |
| Development of future similar studies | - What could possibly motivate you to participate in a more adherent way? - How do you suggest future interventions to be like? |
